# Predicting drug resistance

**DOI:** 10.7554/eLife.103775

**Published:** 2024-11-06

**Authors:** Nicole S Arellano, Shannon E Elf

**Affiliations:** 1 Department of Internal Medicine and the Division of Hematology and Hematologic Malignancies, University of Utah Salt Lake City United States

**Keywords:** leukemic stem cell, CITE-Seq, CML, CD26, CD35, cancer, Human

## Abstract

A new approach helps examine the proportion of cancerous and healthy stem cells in patients with chronic myeloid leukemia and how this influences treatment outcomes.

**Related research article** Warfvinge R, Ulfsson LG, Dhapola P, Safi F, Sommarin MNE, Soneji S, Hjorth-Hansen H, Mustjoki S, Richter J, Thakur RK, Karlsson G. 2023. Single cell multi-omics analysis of chronic myeloid leukemia links cellular heterogeneity to therapy response. *eLife* **12**:RP92074. doi: 10.7554/eLife.92074.

In 2001, a cover from Time magazine showed a handful of yellow pills deemed to be ‘bullets’ in the ‘war against cancer’ (Lemonick and Park, 2001). Inside these pills, a compound called imatinib specifically targets a mutation commonly found in chronic myeloid leukemia (CML), a rare type of cancer that affects bone marrow and blood cells (Druker et al., 1996; Druker et al., 2001; Deininger et al., 2005).

This discovery prompted the development of similar drugs known as tyrosine kinase inhibitors which can treat a variety of cancers, including CML. While these drugs have proven successful, resistance to treatment remains a significant problem.

This is due to the persistence of cancer-initiating cells that can survive treatment and repopulate the eliminated cancer cell population, leading to a reoccurrence of the disease. In CML, these cancer-initiating cells are known as leukemic stem cells and carry a genetic mutation called BCR:ABL. The mutation causes two genes (*BCR* and *ABL*) to fuse and produce a protein that promotes cell division, resulting in excessive amounts of immature blood cells that contain the BCR:ABL mutation and cause the symptoms of CML.

Tyrosine kinase inhibitors for CML, such as imatinib, only target cells that carry the BCR:ABL mutation while leaving healthy cells unscathed (Rowley, 1973). However, some leukemic stem cells have a ‘bulletproof vest’ that protects them from these drugs. Studying leukemic stem cells in blood cancers paves the way to discover how this resistance occurs and how leukemic stem cells can be better targeted in order to be fully eradicated. However, it can be difficult for researchers to quickly and easily distinguish leukemic stem cells from healthy stem cells in bone marrow, particularly hematopoietic stem cells which develop into the cell types found in blood.

Another reason studying leukemic stem cells is challenging is due to a phenomenon called cancer heterogeneity, whereby cells within the same population carry unique sets of mutations that result in varied behaviors that can alter responsiveness to treatment (Fisher et al., 2013; Figure 1). Heterogeneity is frequently seen in leukemic stem cells and is the driving mechanism for resistance (Yanagisawa et al., 2016). Understanding this heterogeneity requires examining both the genes that cells activate and the proteins present on their surface. Now, in eLife, Ram Krishna Thakur, Göran Karlsson from Lund University and coworkers – including Rebecca Warfvinge (Lund University) as first author – report how they used a method called CITE-seq, which records both these factors in a single cell simultaneously, to evaluate the composition of stem cells in CML patients (Warfvinge et al., 2023).

**Figure 1.**
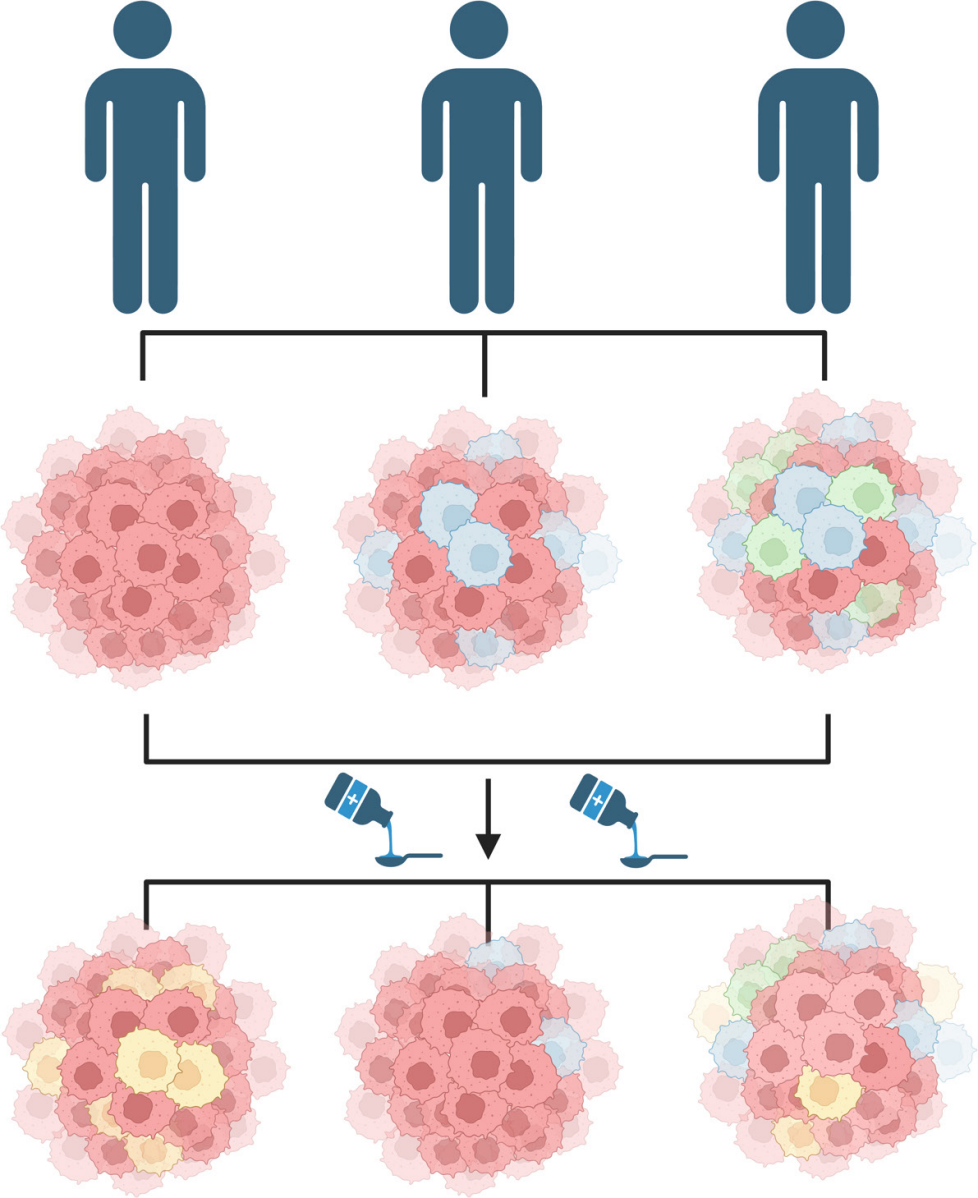
Diversity of cancer cells can influence treatment outcomes. Patients diagnosed with the same cancer (blue figures) may have varied compositions of cancer-initiating cells (top). For instance, the cancer-initiating cells may carry different sets of genetic mutations and/or express different proteins on their surface resulting in subpopulations (denoted by different colors). This diversity may cause the patients to respond differently to the same treatment (bottom). Some patients may grow a new subpopulation of cancer cells in response to treatment (left figure), while the treatment may eliminate an already existing subpopulation in other patients (middle figure), or cause a combination of both effects (right figure). This figure was created with BioRender.com.

The team (who are based at various institutes in Finland, Norway and Sweden) applied CITE-seq to bone marrow samples from nine patients before and 12 months after they had been treated with a tyrosine kinase inhibitor. This multi-pronged detection method allowed Warfvinge et al. to delineate healthy and cancerous stem cells more easily, and see how the composition of stem cells present in a patient’s sample correlated with how well they responded to treatment.

The analysis revealed that leukemic and hematopoietic stem cells express two surface proteins – called CD26 and CD35 – to varying degrees. Specifically, leukemic stem cells which were positive for the BCR:ABL mutation expressed high levels of CD26 but low levels of CD35. Meanwhile, healthy hematopoietic stem cells lacking the BCR:ABL mutation showed the opposite pattern, and expressed low levels of CD26 and high levels of CD35.

Further experiments revealed that levels of CD26 and CD35 could be used to separate and measure the number of leukemic and hematopoietic stem cells in patient samples. Using this approach, Warfvinge et al. were able to show a striking connection between the proportions of these two cell populations and treatment outcomes: patients with a higher percentage of leukemic stem cells at diagnosis were more prone to treatment failure, whereas patients with elevated levels of hematopoietic stems cells were more likely to respond positively to the drug.

The work of Warfvinge et al. could make it easier for physicians to analyze the composition of stem cells in the bone marrow of patients diagnosed with CML. This could help them make more informed predictions about how a patient will respond to tyrosine kinase inhibitors, and develop a treatment plan that works best for them.

In the future, these finding could also help researchers develop personalized treatment strategies for other types of cancer. Treatment strategies should be tailored to the specificities of each person’s cancer, and the work of Warfvinge et al. brings us one step closer to achieving this goal.
